# A Highly Sensitive and Selective Hydrogen Peroxide Biosensor Based on Gold Nanoparticles and Three-Dimensional Porous Carbonized Chicken Eggshell Membrane

**DOI:** 10.1371/journal.pone.0130156

**Published:** 2015-06-12

**Authors:** Di Zhang, He Zhao, Zhuangjun Fan, Mingjie Li, Penghui Du, Chenming Liu, Yuping Li, Haitao Li, Hongbin Cao

**Affiliations:** 1 Beijing Engineering Research Center of Process Pollution Control, Key Laboratory of Green Process and Engineering, Institute of Process Engineering, Chinese Academy of Sciences, Beijing 100190, China; 2 Key Laboratory of Superlight Materials and Surface Technology, Ministry of Education, College of Material Science and Chemical Engineering, Harbin Engineering University, Harbin, China; 3 Environmental Protection Research Institute of Light Industry, Beijing Academy of Science and Technology, Beijing, China; Vrije Universiteit Brussel, BELGIUM

## Abstract

A sensitive and noble amperometric horseradish peroxidase (HRP) biosensor is fabricated via the deposition of gold nanoparticles (AuNPs) onto a three-dimensional (3D) porous carbonized chicken eggshell membrane (CESM). Due to the synergistic effects of the unique porous carbon architecture and well-distributed AuNPs, the enzyme-modified electrode shows an excellent electrochemical redox behavior. Compared with bare glass carbon electrode (GCE), the cathodic peak current of the enzymatic electrode increases 12.6 times at a formal potential of −100mV (vs. SCE) and charge-transfer resistance decreases 62.8%. Additionally, the AuNPs-CESM electrode exhibits a good biocompatibility, which effectively retains its bioactivity with a surface coverage of HRP 6.39×10^−9 ^mol cm^−2^ (752 times higher than the theoretical monolayer coverage of HRP). Furthermore, the HRP-AuNPs-CESM-GCE electrode, as a biosensor for H_2_O_2_ detection, has a good accuracy and high sensitivity with the linear range of 0.01–2.7 mM H_2_O_2 _and the detection limit of 3μM H_2_O_2 _(S/N = 3).

## Introduction

Horseradish peroxidase (HRP), a heme-containing enzyme, is commonly applied for amperometric biosensors due to its redox activity [1, 2]. In particular, enzymatic biosensors, with high stability, accuracy and operability, are superior to those modified by chemical methods. A HRP molecular (42 kDa), is characterized by 308 amino acid residues with a ferric heme (iron protoporphyrin type IX) prosthetic group. It plays an important role in the activation of substrate and the subsequent redox reaction [2, 3]. Recently, the heterogeneous direct electron transfer (DET) between the redox sites of enzyme and the electrode, which is the basis for fabricating various amperometric biosensors and understanding the redox process, has drawn more attention [4, 5]. Nevertheless, since most of the enzymes’ redox centers are deeply buried into their structure, DET barely occurs with the enzymes. Thus, the low rates for electron transfer could limit the application of enzymatic biosensors [6]. In order to enhance DET on the enzymes, electron transfer promoters are widely employed [7].

Over the years, numerous materials, such as gold nanoparticles (AuNPs) [8], carbon nanotubes [9], conducting polymer [10], and biopolymer chitosan [11] have been applied to the modification of the electrode for accelerating electron transfer between the redox center of HRP and electrode surface. Among them, AuNPs have attracted interests owing to their good electrical conductivity, large specific surface area and strong ability to adsorb proteins with preserved bioactivity [12]. Furthermore, AuNPs with high biocompatibility could simulate the environments of redox proteins in a natural system and allow the proteins assume preferred orientation to reduce resistance and promote electron transfer as conduction center [13–15].

Recently, doped carbon has been commonly used in biosensors as a result of its larger functional surface area, more biocompatible microenvironment and higher electrical conductivity [16, 17]. Some researchers reported carbonizing renewable biomass is more effective to enhance electrochemical properties [18–20]. Oxygen and nitrogen molecules in the carbon frame of biomasses could possess a critical role in electrode performance by providing the necessary pseudo-capacitance [21]. Thanks to the unique three-dimensional porous structure, carbonized eggshell membranes (CESM) can achieve a high capacitance of 284–297 F·g^-1^ when used as supercapacitor materials [22]. Besides, due to its excellent biocompatibility and electron transfer property, eggshell membrane (ESM) has also been employed as a template for the synthesis of nanoscale materials used in adsorbents [23] and immobilization supports [24]. Considering the fact that over 1,000 billion eggs are consumed worldwide annually, and each egg can generate 30–40 mg carbon, the eggshell membrane is indeed a reliable and sustainable material for energy storage and sensors. However, enzyme immobilized on the CESM as a modified carbon electrode, especially for metal nanoparticles used as a facilitator, has not been studied yet. Furthermore, whether the metal nanoparticles on the carbonized CESM structure can facilitate DET between enzyme and electrode surface still need to be clarified in future.

In this study, CESM supported on glassy carbon discs electrode with deposition of AuNPs was synthesized and used as a HRP biosensor for detecting H_2_O_2_. The physical structure and chemical composition of the modified electrodes have been characterized by using scanning electron microscope (SEM) equipped with energy dispersive X-ray spectrometer (EDS), X-ray diffraction (XRD), Fourier-transform infrared spectroscopy (FTIR), and X-ray photoelectron spectroscopy (XPS). Furthermore, the electrochemical redox behaviors of the modified electrodes were experimentally estimated by using cyclic voltammetry, electrochemical impedance spectroscopy, and chronoamperometry (i-t). Finally, the electrocatalytic response of the immobilized HRP electrode towards the reduction of H_2_O_2_ was evaluated from the amperometric current-time response. Based on the results, the detection limit and the calibration curve for H_2_O_2_ detection were determined.

## Materials and Methods

### 2.1. Reagents

HRP (EC 1.11.1.7) was obtained from Sigma (USA). Polishing slurries (10 wt.% aqueous suspensions of 0.5 μm and 50 nm α-Al_2_O_3_) were purchased from Tianjin Aida Hengsheng Technology Co. Ltd. (Tianjin, China). Phosphate buffer solution (PBS) was prepared by mixing stock solution of 0.1M KH_2_PO_4_, 0.1M K_2_HPO_4_, NaCl and KCl and adjusting the pH with 0.1M H_3_PO_4_ or 0.1M NaOH. All other chemicals used in this study were of analytical grade and were obtained from Sinopharm Chemical Reagent Beijing Co., Ltd (China). All the solutions used in this study were prepared in ultra-pure water (18.2 MΩcm^-1^, Milli-Q, Millipore).

### 2.2. Preparation of CESM-modified electrode

The ESM was obtained by etching away the hard eggshell (mainly CaCO_3_) with forceps and cleaned with ultra-pure water. The as-obtained ESM was placed on a 1×1 cm^2^ glass carbon electrode (GCE), dried in air, followed by heating in a laboratory oven at 80°C for 4–5h. Subsequently, the ESM was carbonized at 800°C for 2 h in a tube furnace under N_2_ flow at the rate of 100 mL min^-1^. The heating rate was 10°C min^-1^. Upon carbonization, the ESM was converted into a uniform carbon film that combined closely with the surface of the carbon disc to form CESM-GCE. In our experimental conditions, after carbonization, the CESM yield from ESM is 45% ± 5%.

### 2.3. Preparation of enzyme-modified electrode

**Fig 1:**
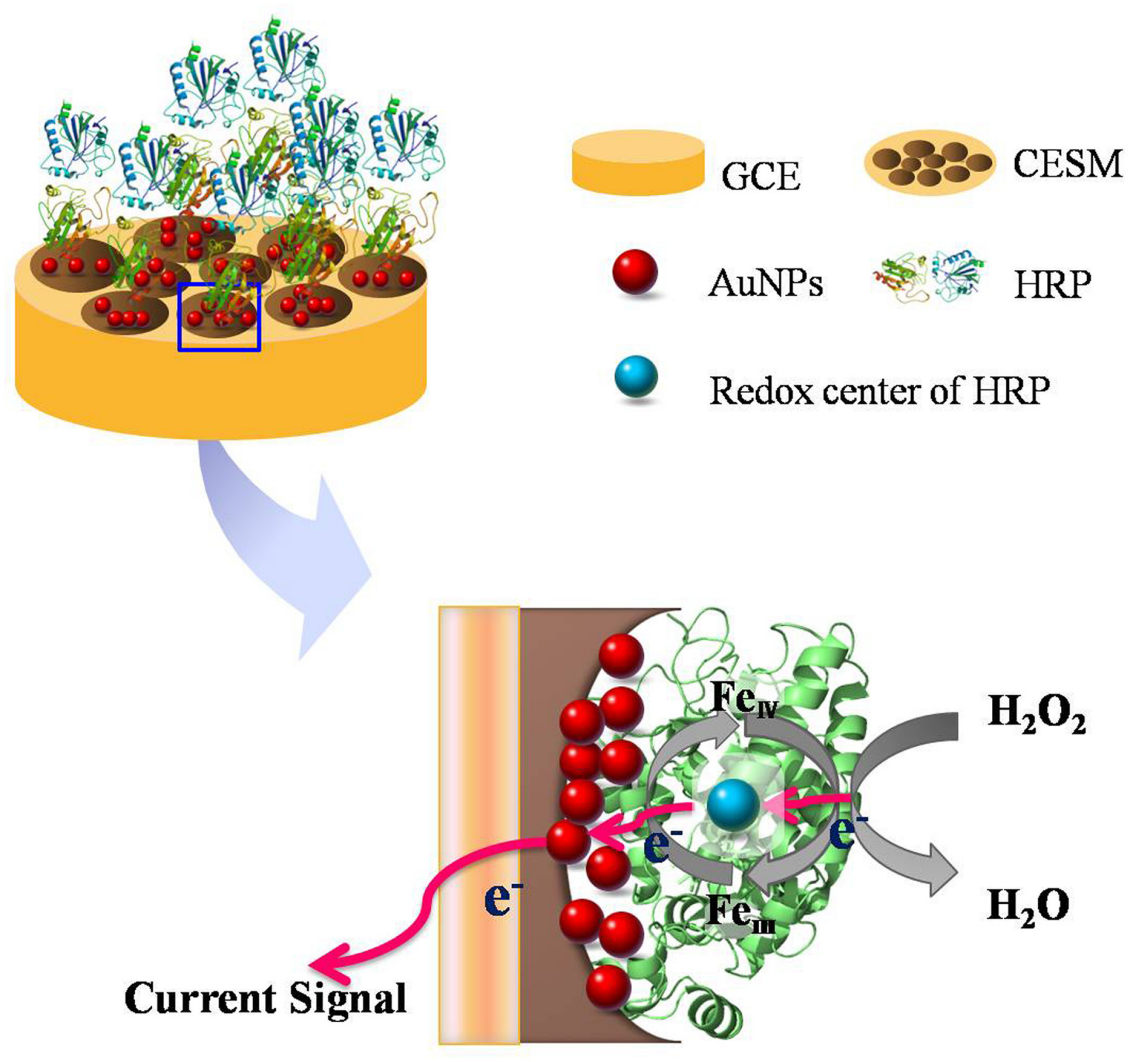
Schematic illustration of the mechanism underlying the detection of H_2_O_2_ using HRP-AuNPs-CESM-GCE electrochemical biosensor.

Electrodeposition via chronoamperometry was performed in 1% (w/w) HAuCl_4_ solution at a constant current of -1mA for 60 s to form a nano-Au layer. Following the electrodeposition, the nano-Au layer was washed in ultra-pure water to form AuNPs-CESM-GCE modified electrode. Then 20 μL of HRP solution (5 mg/ml, 0.1 M pH 7.0 PBS) was dropped onto the surface of the CESM-GCE using a micro syringe. The resulting HRP-AuNPs-CESM-GCE was dried at room temperature, followed by vigorous rinsing in 0.1 M PBS (pH 7.0) to wash out the residual free HRP from the electrode surface. The enzyme-modified electrode thus obtained was used for electrochemical investigations or stored in a refrigerator at 4°C when not immediately used. The schematic illustration of preparation for the hydrogen peroxide biosensor is shown in Fig 1.

### 2.4. Physical and chemical characterization

The morphology and microscopic structure of CESM, and CESM-modified GCEs were characterized by using SEM and EDS (Thermal Field Emission-SEM, JSM-7001F) at an accelerating voltage of 5 kV. The FTIR spectra of the samples were recorded on a Spectra GX spectrometer (PerkinElmer, USA) operating under the transmittance mode. The FTIR spectra were acquired in the wavenumber range of 400–4000 cm^-1^ at the resolution of 1 cm^-1^. The XRD patterns were recorded on PANalytical X-ray diffraction system (Empyrean, Netherlands). XPS data were obtained on an electron spectrometer (ESCALab 250Xi, VG Scientific, Britain) using Al Kα radiation at the power of 300 W.

### 2.5. Electrochemical characterization

The electrochemical characteristics of the modified electrode were characterized by cyclic voltammetry (CV), electrochemical impedance spectroscopy (EIS), and chronoamperometry (i-t) measurements in a conventional electrochemical cell containing a three-electrode arrangement. The electrochemical work station used for this purpose (Autolab PGSTAT 302N, Metrohm China Co. Ltd., Switzerland) is equipped with Nova 1.9 software. Bare glass carbon electrodes (GCEs, 1cm×1cm×1mm, Aida Hengsheng Technology Co., Ltd., Tianjin) were used as base electrodes. Before modification, the bare GCE was polished successively in α-Al_2_O_3_ polishing slurries with 0.5 μm and 50 nm until the surface of GCE was as smooth as a mirror. The well-polished GCEs were then rinsed with ultra-pure water, and ultrasonicated in ethanol and ultra-pure water for 5 min, respectively.

The cyclic voltammograms were recorded in the range of −0.7 V to 0.5 V at a scan rate of 50 mV s^-1^. The Nyquist plots obtained from EIS analysis were recorded over the frequency range of 0.1 Hz to 10^5^Hz with amplitude of 10 mV, and were subsequently superimposed on the open circuit potential of each modified electrode. The electrolytic cell and all the conducting wires were shielded with a copper wire mesh to prevent electromagnetic interference. Evaluation and simulation (SIM) were performed using the ZSimpWin software. The steady-state current of the amperometric i-t curve under constant potential of −0.2V was used for quantitative analysis.

The electrolyte consisted of 0.1M PBS and 1.0 mM [Fe(CN)_6_]^3-/4-^ (1:1 mixture) in 0.1M PBS solution. Bare and modified GCEs were used as working electrodes. A platinum electrode (10 mm × 10 mm × 0.1 mm) was used as the counter electrode, and a saturated calomel electrode (SCE) was used as the reference electrode. The reported potential values are in reference to SCE. All the electrochemical measurements were performed at room temperature. Prior to electrochemical measurements, the buffer solutions were purged with nitrogen (99.99% purity) for 15 min. The nitrogen environment was maintained over the solutions in the cell to protect the solution from oxidation.

## Results and Discussion

### 3.1. Characterization of modified electrode

**Fig 2:**
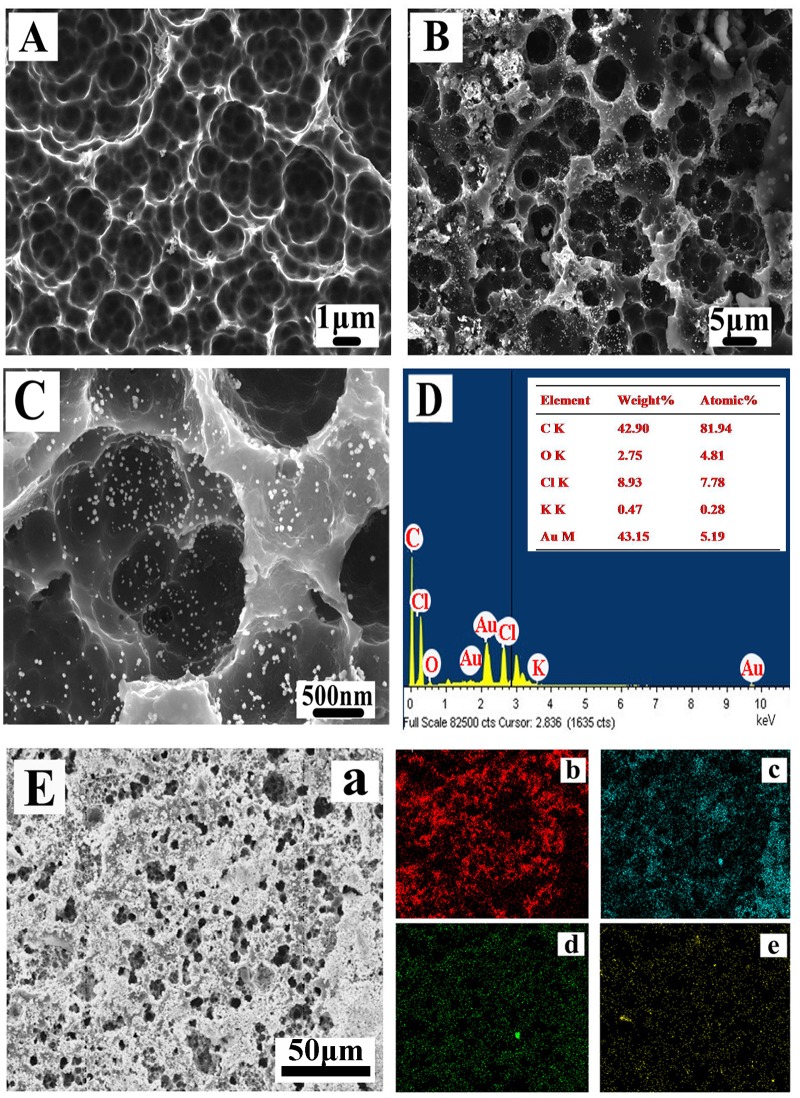
Representative SEM images and EDS elemental analysis of modified electrode. SEM images of CESM-GCE (A) and AuNPs-CESM-GCE (B), (C). EDS elemental analysis (D) and scanning images (E) of AuNPs-CESM-GCE; EDS element scanning image with all elements (a) and elemental distribution of C (b), Cl (c), Au (d) and O (e).

The texture and morphological properties of the modified electrode were observed by electron microscopy scanning (Fig 2). Compared with bare GCE, the CESM-modified GCE is porous upon carbonization of the eggshell membrane (Fig 2A). The membrane is found to be a macroporous network composed of interwoven and coalescing holes with diameters ranging from 0.5 to 5 μm. Furthermore, the SEM images clearly indicate the presence of 1 μm macropores. The surface images of the AuNPs-modified electrode at different magnifications (Fig 2B and 2C) reveal that the surface of the AuNPs-CESM-GCE film is rather rough with numerous well distributed globular nanoparticles on the surface. The size of the nanoparticles is relatively homogeneous and a few dozens of nanometers. The SEM images shown in Fig 2 clearly indicate that the typical structure of the ESM is successfully preserved during the carbonization procedure, and the Au nanoparticles are homogeneously deposited as a result of the electrodeposition process. The macropores and micropores on the carbon plate form a hierarchical porous structure, which are uniformly distributed in the GCE. Such a long-range continuous pore network is critical for realizing a fast electrolyte transfer [25].

Furthermore, the chemical composition of the modified electrodes was analyzed by using EDS. The formation of AuNPs on the CESM-GCE is substantiated by the EDS analysis shown in Fig 2D, which indicates the presence of C, Au, Cl, and O elements in the composite. The C and O peaks in the EDS spectrum could be attributed to the CESM-GCE, acting as a template for electrodeposition of Au. The peak corresponding to Cl is derived from the HAuCl_4_ used in the electrodeposition process. The inset shown in Fig 2D provides a detailed summary of the weight percentage of the elements corresponding to the composite on the modified GCE. Fig 2E displays the EDS elemental mapping of the modified AuNPs-CESM-GCE at the microstructural level. As seen in Fig 2E (d), Au is well distributed on the surface of the sample.

**Fig 3:**
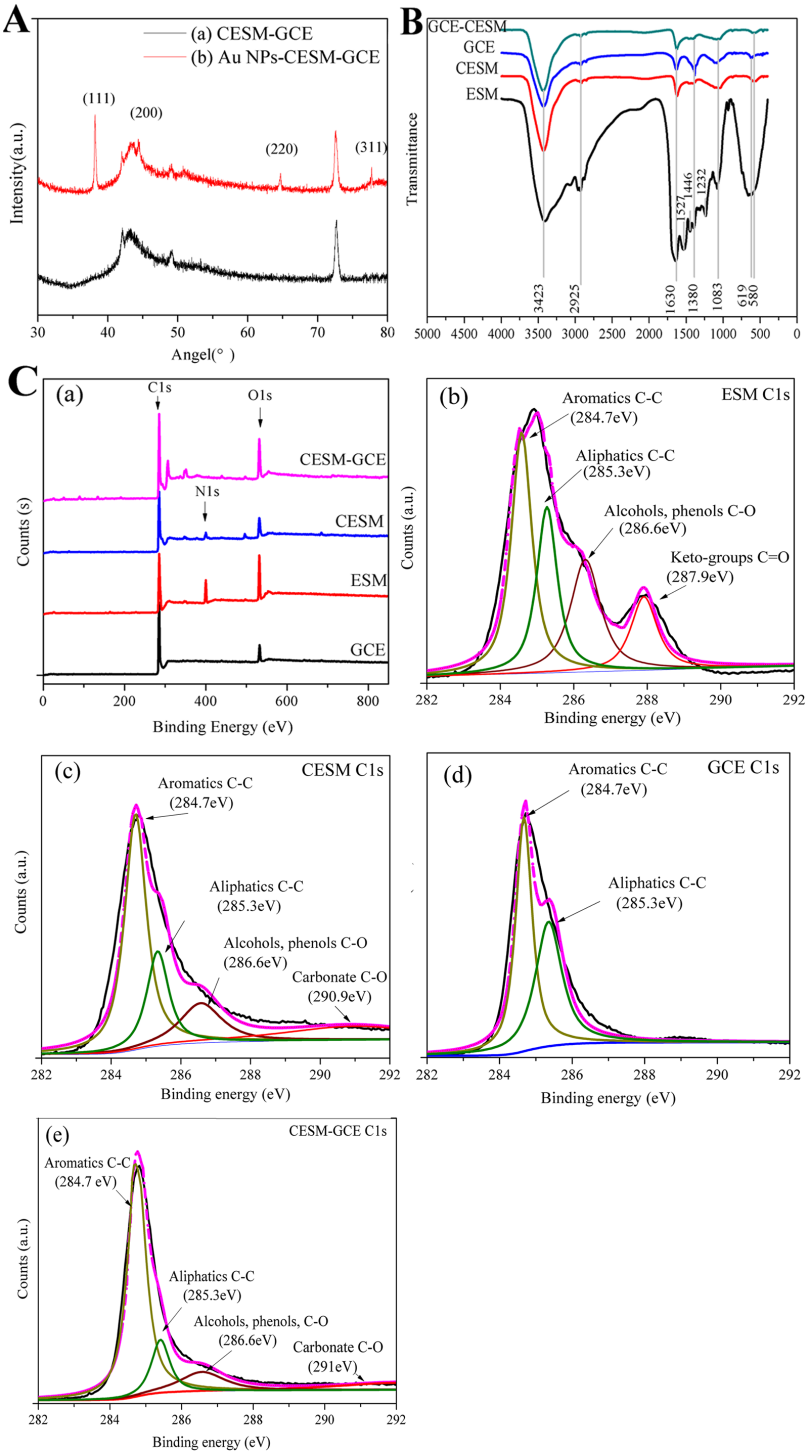
X-ray diffraction patterns (A), FTIR spectra (B) and XPS spectra (C) of modified electrodes. XPS spectra of wide-scan survey (a), XPS C1s spectra of ESM (b), CESM (c), GCE (d) and CESM-GCE (e).

The XRD patterns of the CESM-GCE and AuNPs-CESM-GCE are illustrated in Fig 3A. The diffraction peaks observed at 42.1°, 43.2°, 49.2° and 72.7° in both the samples are corresponded to the characteristic functional groups of the CESM modified carbon (curve a). Considering the diffraction pattern of AuNPs-CESM-GCE, the additional peaks observed at 38.2°, 44.4°, 64.7° and 77.7° could be ascribed to the (111), (200), (220) and (311) planes of the Au crystals (curve b) [25]. These results confirm the formation of AuNPs on the surface of the modified electrode. Besides, the modified electrode shows retained even structure after electrodeposition, which confirms the SEM observations.

ESM, which is mainly composed of protein and polysaccharide compounds, consists of many active groups such as hydroxyl (OH), amino group (NH_2_), carbonyl (-C = O), amide (-CO-NH_2_) [26]. Fig 3B depicts the FTIR spectra of ESM, CESM, GCE, and CESM-GCE. The FTIR spectra of all the four samples are almost similar. The FTIR spectrum of ESM shows peaks at 1527, 1446, and 1432 cm^-1^ corresponding to the presence of amino and amide groups [27]. Besides, the peak at about 580 cm^-1^ in the FTIR spectra of CESM and CESM-GCE, and the peak at around 619 cm^-1^ in the FTIR spectrum of ESM could be associated with the in-plane deformation and out-plane deformation modes of calcium carbonate [27]. The vibrations at 3423, 2925, 1630, 1380 and 1083 cm^-1^ are observed in all the samples, and the absorption band at 3423 cm^-1^ suggests the existence of −OH (coupling ν O-H). The band at 2925 cm^-1^ can be attributed to the CH_3_ or CH_2_(C-H) stretching vibration, and the band at 1630 cm^-1^ should be corresponded to the C = O stretching vibration of the egg membranes polypeptide skeleton. The peak at 1380 cm^-1^ is ascribed to C-O stretching vibration of epoxy groups, and the peak at 1083 cm^-1^ is attributed to C-O stretching vibration of alkoxy groups [28]. These results clearly indicate the presence of oxygen functional groups in the CESM composite film.

The XPS analyses of all the samples are presented in Fig 3C. As shown in Fig 3C (a), the ESM and CESM samples contain C1s, N1s, and O1s peaks, while the N peak is not obvious in the CESM-modified glassy carbon electrode. In addition to the presence of C, N, and O, the CESM-modified GCE also contains other impurities, such as Mg (306.4eV) and Ca (351.4eV). As seen in the wide-scan XPS survey spectra, the carbonization of the ESM results in a 14.4% increase of C content, 6.7% and 7.7% decrease of O and N content, respectively (Table 1). The nitrogen content of CESM-GCE is very low (0.32%). In the process of XPS measurement, the ESM, CESM and GCE are powder samples which were prepared by a tableting process, while the CESM-GCE sample is a carbonized membrane directly distributed in a plate electrode, which couldn’t be prepared in powder form. We know that the typical detection depth of XPS is ~5 nm. So the modified CESM-GCE (electrode) sample is less uniformly distributed than the GCE and CESM samples (powder) during XPS analysis. This may explain why part of the CESM-GCE sample was detected and why the observed atomic percentage of N is lower for the modified CESM-GCE compared to bare GCE and CESM. Nevertheless, the O/C ratio in the CESM-GCE is 0.24, which is 1.71 times of that in the CESM. Since O can generate pseudo-capacitance, the increase in O/C ratio is important for the enhancement of the electrochemical performance of the electrode.

**Table 1:** Elemental composition determined from the wide-scan survey XPS spectra, and sp^2^/(sp^2^+sp^3^) of C1s spectra for ESM, CESM, GCE, and CESM-GCE.

	ESM	CESM	GCE	CESM-GCE
**C(%)**	68.2	82.68	89.93	80.44
**N(%)**	13.54	5.82	1.08	0.32
**O(%)**	18.26	11.5	8.99	19.24
**sp^2^/(sp^2^+sp^3^)**	0.59	0.71	0.55	0.81

Furthermore, the C1s XPS spectra of all samples (Fig 3C (b), (c), (d) and (e)) were fitted to estimate the relative surface concentrations of the functional groups from the corresponding peak sizes. The C1s XPS spectrum of ESM shows the peaks correspond to aromatics (C-C), aliphatics (C-C), alcohols or phenols (C-O), and ketone groups (C = O), respectively. After carbonization, the change of aromatics and aliphatic peaks can be observed, together with the ketone groups (C = O) replaced by carbonates (C-O). According to previous studies, the sp^2^ structure (corresponding to aromatic plane carbon) could facilitate high electrochemical activity for biomolecules and provide better electrochemical performance when compared with sp^3^ hybridized carbon (corresponding to aliphatic crystal carbon) [29]. Therefore, the ratio of sp^2^ to sp^3^ structure is considered to be important for its electron transfer properties. On the basis of these viewpoints, the sp^2^/(sp^2^ + sp^3^) results in Table 1 suggest that sp^2^ proportion in the CESM increases 20.3% when compared to that of ESM. This can imply that the carbonization process has a great impact on the structure of the surface carbide distribution. Furthermore, 14% increase of the sp^2^/(sp^2^ + sp^3^) ratio of the CESM-GCE after modification with GCE, indicates the possible interaction between the surface of the carbon electrode and the eggshell membrane. Thus, a more planar carbon structure, which is conducive for electron transfer, was produced.

### 3.2. Electrochemical behavior of the different film-modified GCE electrode

#### 3.2.1. Cyclic voltammetry characterization

**Fig 4:**
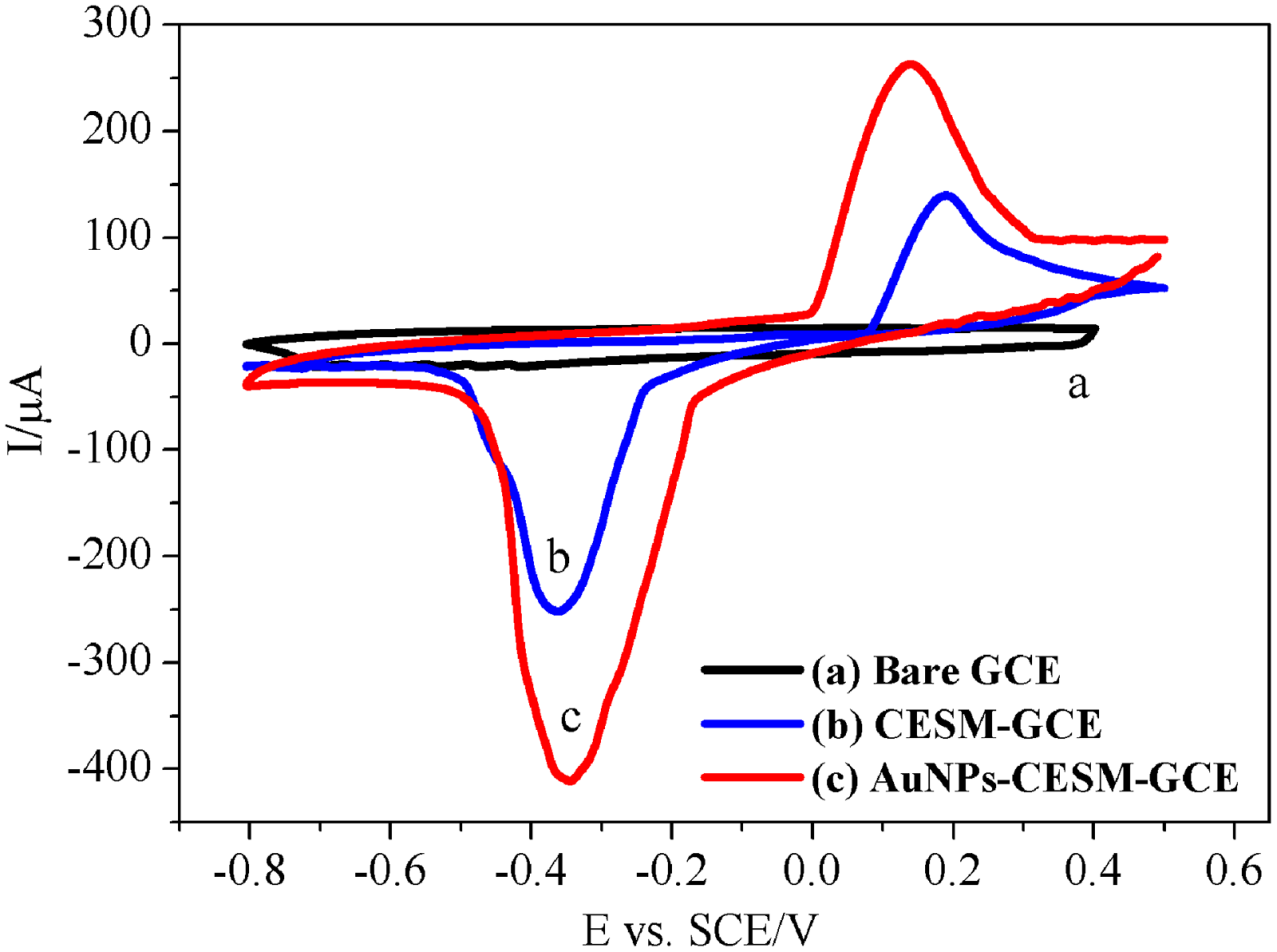
Cyclic voltammograms of modified electrodes. Bare GCE (a), CESM-GCE (b), AuNPs–CESM-GCE (c), in 0.1 M pH 7.0 PBS at a scan rate of 50 mV/s.

Fig 4 shows the cyclic voltammograms of the bare GCE (a), CESM-GCE (b), AuNPs-CESM-GCE (c) in 0.1 M pH 7.0 PBS at a scan rate of 50mV/s. The CV of the bare GCE does not have a redox peak, indicating the absence of redox activity in the selected potential range. On the other hand, the CESM-modified electrode displays a pair of redox peaks (Fig 4b), which can be assigned to the electrochemical oxidation and reduction processes of the CESM. The peak current of the AuNPs-CESM-GCE electrode (Fig 4c) is found to be higher than that of CESM-GCE.

Comparing the current responses of these fabricated electrodes, the enhancement in the current response of CESM-GCE can be attributed to the enhanced surface area in the presence of the CESM. The CESM-GCE electrode provides a large number of active sites for the electrocatalytic process and also binds with HRP for the enzymatic reaction. Given the advantages of the large specific surface area and good conductivity of AuNPs, the AuNPs–CESM-GCE electrode exhibits enhanced current response [30]. These results indicate that the CESM and AuNPs act as promoters for successfully establishing direct electron transfer and dramatically enhance the electrochemical response.

#### 3.2.2. Electrochemical impedance spectroscopy (EIS) characterization

Furthermore, the interfacial properties of the modified electrode, which are vital significant for the electrical conductivity, and the electrocatalytic features of the modified electrode were analyzed by EIS using the redox probe [Fe(CN)_6_]^3-/4-^ [31, 32]. The electron-transfer kinetics and diffusion characteristics can be determined from the shape of the impedance spectrum. The semicircular portion obtained at higher frequencies corresponds to an electron transfer-limited process and the linear portion at lower frequencies is attributed to the limited mass transfer of the ferricyanide ion. The electron transfer characteristics were interpreted by using the Randle’s equivalent circuit [18]. The impedance spectrum of the HRP-AuNPs-CESM-GCE electrode is well fitted with the Randle’s circuit consisting of electrolyte resistance (Rs), electron transfer resistance (Rct), double layer capacitance CPE (C_dl_) and Warburg impedance (W) [13].

**Fig 5:**
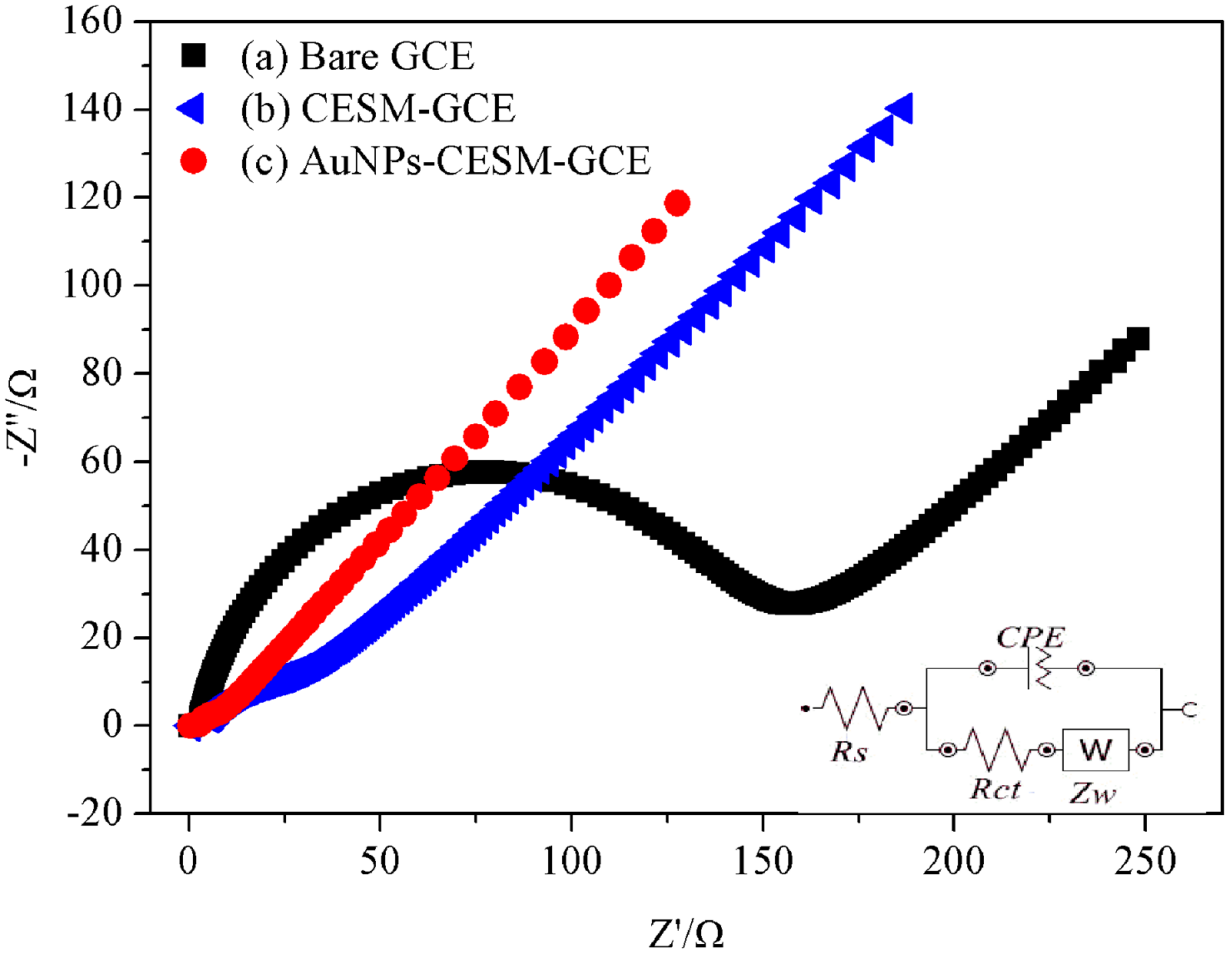
Electrochemical impedance spectroscopy of modified electrodes. Bare GCE (a), CESM-GCE (b), AuNPs–CESM-GCE (c), in 0.1 M pH 7.0 PBS containing 1 mM [Fe(CN)_6_]^3-/4-^ (inset: Randle’s equivalent circuit).

As seen, the shapes of the EIS spectra of the five electrodes are very different during the stepwise assembly of the biosensor. As shown in Fig 5, the Nyquist plot of the bare GCE (Fig 5a) displays a well-defined, enlarged semicircle with an electron transfer resistance (Rct) of about 148.9 Ω at high frequencies (Table 2). Compared with the bare GCE (Fig 5a), the diameter of the semicircle in the Nyquist plot of the CESM-modified GCE decreases dramatically (Fig 5b), featuring a lower Rct of 35.48Ω. The electrode modified by AuNPs film exhibits a much lower resistance of 11.38 Ω (Fig 5c), and the Nyquist plot of the electrode shows a nearly straight line. It is consistent with previous study, which indicates that the AuNPs are an excellent conducting material and could act as tiny conduction centers to promote the electron transfer [33]. The results of the impedance change during the electrode modification, provide an evidence for the successful immobilization of the CESM and AuNPs on the electrodes. These results also prove that the CESM and AuNPs could improve the electron transfer rate between the electrode and immobilized enzymes, which is consistent with the CV results shown in Fig 4.

**Table 2:** EIS data of different modified electrodes in 1 mM [Fe(CN)_6_]^3-/4-^.

Electrode	R_s_(Ω)	CPE(F)	n	R_ct_(Ω)	W(Ω)
**Bare GCE**	2.77	3.71E-05	0.824	148.9	0.0094
**CESM-GCE**	4.17	5.17E-03	0.317	35.48	0.0004
**AuNPs-CESM-GCE**	2.33	3.33E-03	0.484	11.38	0.0072

### 3.3. Hydrogen peroxide response characteristics of the biosensor

**Fig 6:**
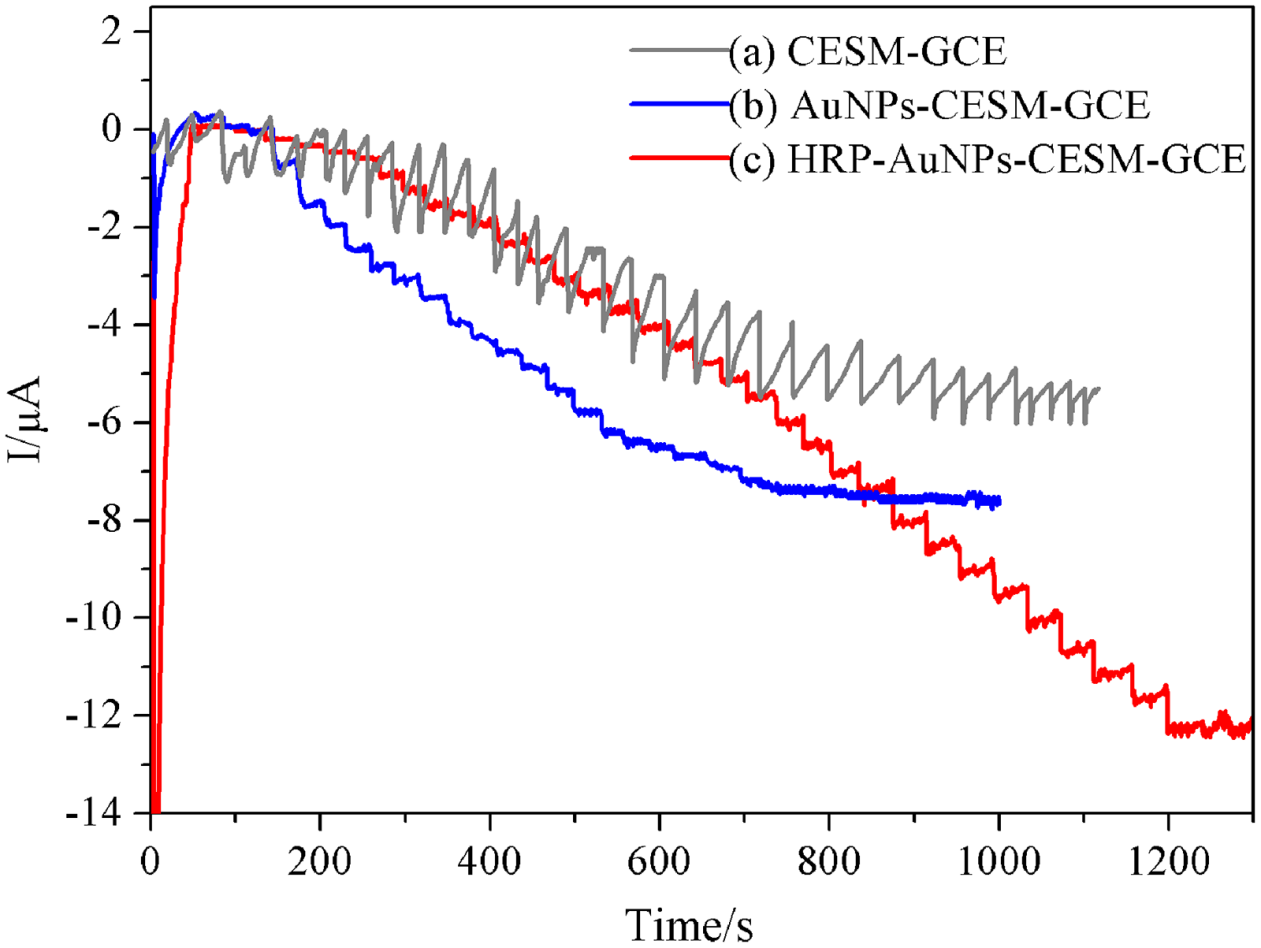
Typical amperometric responses of the different modified electrodes. The applied potential was -0.2 V vs. SCE in 0.1 M PBS solution (pH = 7.0).

**Fig 7:**
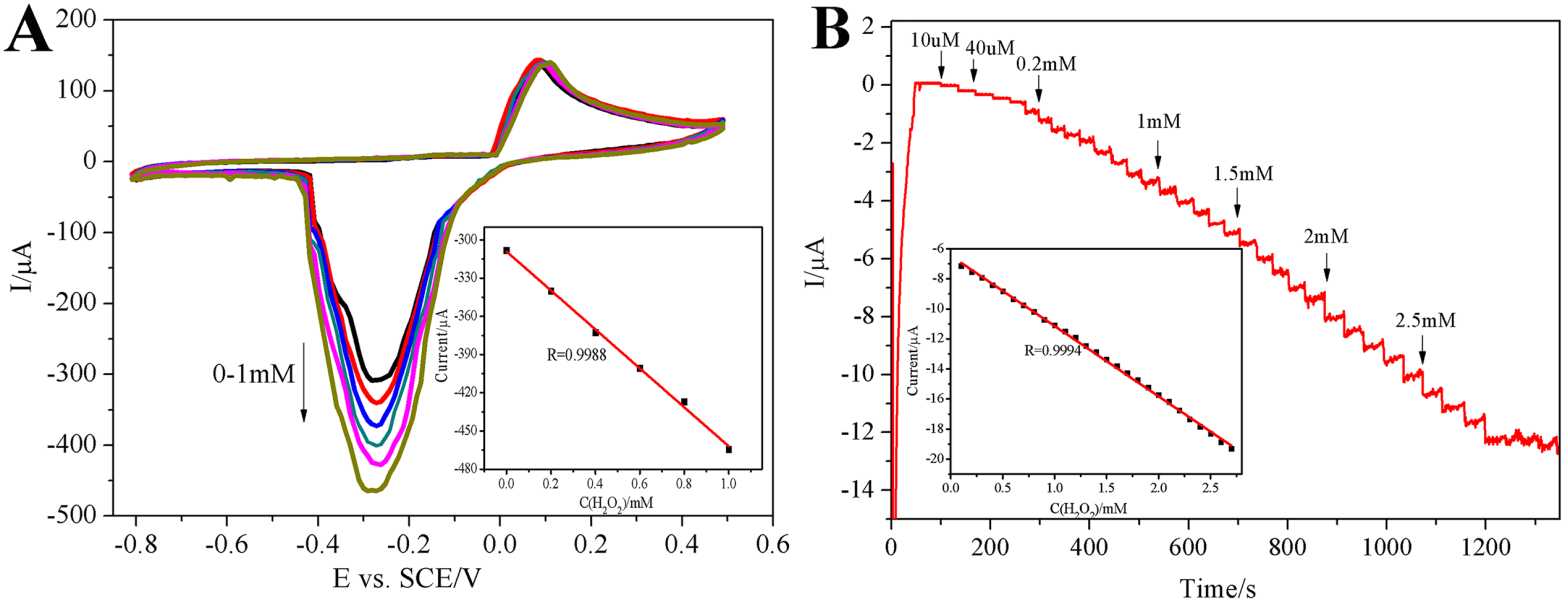
Cyclic voltammograms (A) and amperometric responses (B) of HRP-AuNPs-CESM-GCE electrode with successive addition of H_2_O_2_. (A) 200 μM H_2_O_2_ was added continuously at a scan rate of 50 mV/s (inset: corresponding calibration plot of peak current against different concentrations of H_2_O_2_). (B) Amperometric responses of HRP-AuNPs–CESM-GCE electrode during successive addition of aliquots of H_2_O_2_ (inset: corresponding calibration plot of steady-state currents as a function of different concentrations of H_2_O_2_) at the applied potential of -0.2 V vs. SCE in 0.1 M PBS solution (pH = 7.0).

The amperometric current-time response was recorded to estimate the detection limit and the calibration curve for H_2_O_2_ detection. Fig 6 describes the comparison of the chronoamperometry responses of different modified electrodes in the assembly process with successive injections of H_2_O_2_ to the PBS (pH 7.0) at applied potential of −0.2 V. In case of the CESM-GCE and AuNPs-CESM-GCE electrodes, the reduction current is observed after the addition of H_2_O_2_ (curve a, b), indicating that the CESM-modified electrode is capable of reducing H_2_O_2_. However, the CESM-modified electrode shows unsteady response of reduction current. On the other hand, the AuNPs-CESM-modified electrode exhibits a higher current response, but a worse linear current response towards H_2_O_2_ concentration, with a correlation coefficient of 0.984. Fig 7A shows the influence of AuNPs. As tiny conduction centers, the AuNPs could facilitate the transfer of electrons. In contrast, a larger current and a more steady response is observed by using a HRP-AuNPs-CESM-modified glassy carbon electrode (curve c), revealing that the immobilization of HRP in the modified electrode is essential and sensitive for the detection of H_2_O_2_. Furthermore, curve c clearly demonstrates the response of the resulting biosensor towards H_2_O_2_. Therefore, HRP-AuNPs-CESM-GCE was selected as electrode for H_2_O_2_ determination in our study.

Fig 7A shows the CV response of HRP-AuNPs-CESM-GCE towards H_2_O_2_ at nitrogen saturated PBS (pH 7.0). By addition of 200 μM H_2_O_2_ into the PBS solution, there is a significant increase of the reduction peak at -0.25 V, implying the reduction of H_2_O_2_ at the electrode. The biosensor is capable of detecting H_2_O_2_ at the concentration of 10 μM, suggesting that the effective sensitivity of the biosensor may be due to the presence of HRP, CESM, and AuNPs. Moreover, the calibration curve, corresponding to voltammetric response against the current and concentrations of H_2_O_2_ ranging from 200 μM to 1 mM, follows a linear regression equation as shown below: I (μA) = -309.14–152.82 C_H2O2_(μM); R = 0.9988.

From the results of the average of the anodic and cathodic peaks [25], the formal potential of the HRP-AuNPs-CESM-GCE is calculated to be -100 mV, with a peak potential separation of about 459 mV. This is smaller than that of the HRP-CESM-GCE. Due to the synergistic effects of CESM and AuNPs on the layer, the special interactions could strongly affect the microenvironment of enzymatic active center or the electrode double layer. The formal potential of the HRP-AuNPs-CESM-GCE electrode shifts positively by 95 mV. This result is much higher than the Clay-HRP-Clay/AuCS-GCE electrode (-195 mV) [8] and previous research reported [30, 31], which suggests that the HRP entrapped in the AuNPs-CESM-GCE film undergoes a fast electron transfer process.

According to the integration of reduction peak currents and Faraday’s law, the surface concentration of electroactive HRP (*τ*) in HRP-AuNPs-CESM-GCE surface could be estimated as followed equation:

$$I_{p}=\frac{n^{2}F^{2}A\tau v}{4RT}$$ (1)

where *I*_*p*_ is the reduction peak current, *I*_*p*_ = 3.00×10^-4^ A; *A* is the electrode surface area, *A* = 1 cm^2^; *v* is the scan rate, *v* = 0.05 V s^-1^; *n* is the number of electron, *n* = 1; *F* = 96,485 C mol^-1^; *R* = 8.314 J K^-1^ mol^-1^; and *T* = 298 K. Accordingly, the τ value is calculated to be 6.39×10^-9^ mol cm^-2^, which is about 752 times higher than the theoretical monolayer coverage (8.5×10^-12^mol cm^-2^) of HRP [19]. Comparing the calculated value of τ with the previous reported values for HRP surface concentration in other immobilized matrix, such as nano-Au/choline (1.2×10^-9^ mol cm^-2^) [30] and AuNPs–SF composite (1.8×10^-9^ mol cm^-2^) [21], the AuNPs-CESM composite is highly efficient in immobilizing HRP. Consequently, a large number of HRP molecules are firmly adsorbed onto AuNPs. As a result, the CESM and AuNPs dramatically enhance the electrochemical response of HRP, thereby accelerating the regeneration of the enzyme, and enhancing its bioactivity.

Fig 7B shows the typical amperometric responses of the HRP-AuNPs-CESM-GCE electrode towards H_2_O_2_ at the applied potential of −0.2 V in 0.1 M phosphate buffered saline (pH 7.0). With H_2_O_2_ concentration increase, the amperometric response of the enzyme electrode also increase. Almost 95% catalytic current reaches steady state within 3 sec of injecting H_2_O_2_. The inset shown in Fig 6B reveals the calibration curve of the enzyme electrode at optimum conditions. The biosensor responds to H_2_O_2_ in the linear range from 1×10^-5^ mol L^-1^ to 2.7×10^-3^ mol L^-1^, with a correlation coefficient of 0.9994 and a detection limit of 3×10^-7^ mol/L at a signal-to-noise ratio of 3. It can be seen that the HRP-AuNPs-CESM-GCE electrode offers a reasonable linear range towards the detection of H_2_O_2_. The detection limit is lower than that reported in some previous studies [18, 30]. Moreover, the obtained biosensor exhibits very good accuracy and high sensitivity, indicating an excellent platform for the detection of H_2_O_2_. The proposed method is economical and efficient, making it potentially attractive for real-time sample analysis.

### 3.4. Reproducibility and stability of the biosensor

The repeatability and stability of the HRP biosensor were investigated by measuring the current response of 0.5 mM H_2_O_2_ in pH 7.0 PBS solution. The relative standard deviation (RSD) for 10 successive assays is 3.08% (n = 10), which displays an acceptable reproducibility. The fabrication reproducibility of four HRP enzyme electrodes shows a 5.9% RSD for cyclic voltammetry determination at 0.5 mM H_2_O_2_. Also, the proposed biosensor shows excellent long-term stability. When not in use, the biosensor was stored dry at 4°C, the biosensor retained about 95.4% of its initial response to H_2_O_2_ after 10 days and 88.6% after 20 days. Good long-term stability could be attributed to the strong interaction between HRP and AuNPs embedded in CESM structure, which prevent the loss of enzymatic activity. AuNPs-CESM composite matrix could provide a biocompatible microenvironment. Therefore, the biosensor in our work has good stability and repeatability.

## Conclusions

This study prepared a HRP-AuNPs-CESM-GCE electrode for the detection of H_2_O_2_, via the deposition of AuNPs onto a glassy carbon HRP electrode with carbonized eggshell membrane support. Systematic characterization of the fabricated samples indicates the uniform distribution of AuNPs anchored on a 3D conductive carbon film with the interconnected pore network that facilitates the electron transport between HRP and the electrode surface. Compared with bare GCE, the enzyme-modified electrode shows an excellent electrochemical redox behavior, featuring an increase in cathodic peak current by 12.6 times at a formal potential of −100 mV (vs. SCE) and a decrease in charge-transfer resistance by 62.8%. In addition, the HRP-AuNPs-CESM-GCE electrode, as a biosensor, exhibits a dynamic range between 10 μM and 2.7 mM, with a detection limit of 3 μM and a response time of 3 s towards the detection of H_2_O_2_. The results presented in this study demonstrate a simple method for developing a new class of electrochemical biosensors for H_2_O_2_ detection, with the advantages of low-cost, ease of construction and use, rapid response, and convenient operation.
